# Items

**Published:** 1883-06

**Authors:** 


### Items.

AMERICAN MEDICAL ASSOCIATION.

The Thirty-fourth Annual Session will be held in Cleveland, Ohio, on Tuesday, Wednesday, Thursday and Friday, June 5, 6, 7, 8, 1883, commencing on Tuesday, at 11 A. M.

"The delegates shall receive their appointment from permanently organized State Medical Societies, and such County and District Medical Societies as are recognized by *representation in their respective State Societies*, and from the Medical Department of the Army and Navy, and the Marine Hospital service of the United States.

"Each State, County and District Medical Society entitled to representation shall have the privilege of sending to the Association one delegate for every ten of its regular resident members, and one for every additional fraction of more than half that number: *Provided*, however, that the number of delegates for any particular State, territory, county, city or town shall not exceed the ratio of one in ten of the resident physicians who may have signed the Code of Ethics of the Association."

Secretaries of Medical Societies as above designated are earnestly requested to forward, *at once*, lists of their delegates.

Also, that the Permanent Secretary may be enabled to erase from the roll the names of those who have forfeited their membership, the Secretaries *are, by special resolution*, requested to send to him annually a corrected list of the membership of their respective societies.

SECTIONS.

"The Chairmen of the several Sections shall prepare and read, in the general sessions of the Association, papers on the advances and discoveries of the past year in the branches of science included in their respective Sections. * * *"—*By-Laws*, Art. 11, Sect. 4.

Practice of Medicine, Materia Medica, and Physiology—Dr. J. H. Hollister, Chicago, Ill., Chairman; Dr. J. G. Lee, Philadelphia, Secretary.

Obstetrics and Diseases of Women and Children—Dr. J. K. Bartlett, Milwaukee, Wis., Chairman; Dr. G. A. Moses, St. Louis, Mo., Secretary.

Surgery and Anatomy—Dr. W. F. Peck, Davenport, Iowa, Chairman; Dr. P. F. Eve, Nashville, Tenn., Secretary.

State Medicine—Dr. Foster Pratt, Kalamazoo, Mich., Chairman; Dr. T. L. Neal, Dayton, Ohio, Secretary.

Ophthalmology, Otology and Laryngology—Dr. A. W. Calhoun, Atlanta, Ga., Chairman; Dr. Carl Seiler, Philadelphia, Secretary.

Diseases of Children—Dr. R. F. Blount, Wabash, Ind., Chairman; Dr. J. H. Sears, Waco, Texas, Secretary.

Oral and Dental Surgery—Dr. D. H. Goodwillie, New York City, Chairman; Dr. T. W. Brophy, Chicago, Ill., Secretary.

A member desiring to read a paper before any Section should forward the paper, or its *title* and *length* (not to exceed twenty minutes in reading), to the Chairman of the Committee on Arrangements at least one month before the meeting.—*By-Laws.*

Committee of Arrangements—Dr. X. C. Scott, 393 Euclid Avene, Cleveland, Ohio, Chairman.

Amendments to the Constitution—Offered by Dr. N. S. Smith, Dakota:

"To provide for the admission to membership of two delegates from the Medical Bureau of the United States Indian Service, to be nominated by the Surgeon-in-Chief of the Bureau, and approved by the Secretary of the Interior."

Offered by Dr. J. M. Toner, D. C.:

"That the office of Permanent Secretary be vacated, and that the Nominating Committee hereafter annually nominate a Secretary who will serve without compensation."

Offered by Dr. F. Pratt, Mich.:

"That the law requiring the nominations for officers to be made

*from those members present at the annual meeting*, shall apply only to the President, Vice-Presidents, Chairmen and Secretaries of Sections, the Assistant Secretary, the Chairman of the Committee of Arrgngements, and the Judicial Council."

Offered by Dr. J. M. Keller, Ark.:

"To permit the holding of the annual meeting as late as the first Tuesday of September, if desirable."

Offered by Dr. J. H. Sears, Ark.:

"That the Chairman and Secretary of each Section may add any number of earnest workers to their Sections, in addition to those named by the Nominating Committee, and that the Librarian be made a permanent officer."

Amendments to the By-Laws—Offered by Dr. J. W. Smith, Iowa. Art. II. Sect. 8. Permanent Members: strike out the words "but without the right of voting."

WM. B. ATKINSON, M. D.
Permanent Secretary.

Philadelphia, 1400 Pine st., S. W. cor. Broad.

---

COOK COUNTY HOSPITAL. Reported by DR. E. P. DAVIS.

The interesting operations of the hospital surgeons during the six weeks ending May 15 have been as follows:

Two secondary amputations of the thigh after excision of the knee joint; in one case the primary operation was defeated by secondary hæmorrhage, which necessitated turning back the flaps and the application of styptics; in the other case, that of a child, sinuses were left which refused to close. In both cases, marked improvement followed the second operation.

Amputation of the thigh for rupture of the popliteal artery. The patient, a middle-aged woman, was admitted with distension of the knee joint with pus; limb flexed at right angles and not anchylosed; a history of rigors and sweats. As soon as possible, an attempt was made to straighten the limb and drain the joint freely. Under the exercise of very moderate force, the popliteal artery ruptured. Amputation was immediately performed. The severed joint was found extensively eroded, while pus had burrowed below the knee. Although the tissues above the knee were much impaired in nutrition, the patient's progress has been unexpectedly good.

A large cyst of the parovarium was removed from a woman of middle age. A diagnosis of ovarian cyst was made, but the real nature of the case became apparent on opening the abdomen. To remove the tumor, an extensive dissection of the peritoneum was necessary. A pedicle was made, and cauterized with thermo-cautery. Silk and catgut were used as ligatures, and antiseptic precautions observed. A drainage tube was introduced, and the external wound closed with silk. Death from shock followed three days after the operation. A post-mortem revealed no peritonitis and no hæmorrhage; union of the external wound with inconsiderable suppuration. The application of heat by means of the passage of water through flexible metal tubing, coiled to fit the abdomen, was employed with good results during the case.

An adult male was admitted with an injury to the hip joint, produced by falling backward from a fence about twenty feet. A diagnosis of dislocation into the thyroid foramen was made, crepitus indicating some injury to the acetabulum. Reduction was effected by manipulation without difficulty.

A large epithelioma, involving the entire cheek and chin, and also the inferior maxilla, was removed, and a portion of the jaw at the symphysis exsected. The floor of the mouth was exposed, the submaxillary and sublingual glands removed, and involved lymphatics. Free dissection of the alveolar tissues enabled the operator to close the wound with plastic pins; iodoform was used freely in dressing. The recent occurrence of the operation does not indicate the result at present. The patient's constitution is good.

THE Prospectus of the journal of the American Medical Association will be found on another page. It is understood that its editor will be Dr. N. S. Davis, of Chicago, and that the journal will be published in the same city. This adds a most important influence towards elevating the medical standard of the Mississippi Valley and of the West. But with such a trust in our hands goes also a certain responsibility. If the profession of this part of the nation are wise, they will lend their support in every possible way, not only to this, but to every one of their respectable home journals. In no other way can the medical centers of the West hope to secure for themselves what justly belongs to them. Every year a large number of very valuable contributions, produced by Western men, is sent to Eastern journals. Clearly such a course helps to build up the medical centers of the East, by giving to them what naturally belongs to the West. It has long been a mystery why men who could directly profit by the development of their own sections, as medical centers, are constantly adding to the attractions of distant cities, and to the advantage of those there practicing. It is not denied that whatever adds to the strength of a medical center draws patients to that center, and it follows that when a good, original contribution is sent to New York or Philadelphia, from Chicago or St. Louis, the writer, by his influence, sends, indirectly, with that contribution, valuable professional business that would otherwise more naturally be attracted to his own field. If our medical authors would consider their own interests a little more, and be governed a little less by the traditions of the past, traditions which no longer need anply, they must certainly see that they have not properly appreciated their own opportunities. The *Review* is not asking for itself. It has no grievance, but it would take this occasion to ask in behalf of the new enterprise the hearty co-operation of the whole profession. The Association journal starts with fortunate prospects. Its editor has been well chosen. No man in the profession has done more honest, hard, downright telling work. He now does the profession a favor in undertaking the management of this journal. Its establishment in our midst is a compliment to ourselves. Let us deserve the compliment.—*The Weekly Medical Review.*

BANQUET AT THE ACADEMY OF MEDICINE.—After the addresses of the retiring and incoming presidents (which were received with great fortitude) the members and invited victims enjoyed the following menu:—

SOUP.

Vermicelli, Mutton-Broth, Oatmeal Gruel, St. Ignatius' Bean Soup, Pea-Soup (Alkaline reaction).

FISH.

Cod, with Whale Oil; Morrhuae Salad, Cat, with Caper Sauce, Dog-Fish, Star-Fish.

MEATS.

Nutmeg Liver, Amyloid Kidney, Fatty Heart, Spleen (*Ad Libitum*).

RELISHES.

Pepsin, Ingluvin, Hydro-Piper, Mustard Plasters, Salts, (Glaubers and Epsom), Vinegar (Acetum Opii).

WINE AND LIQUORS.

Vin Ferri, Vin Colchici, V. Aloes, V. Antimon., Liquor Potass., Liq. Arsen., Gin-Seng.

FRUITS.

Adam's Apples, May Apples, Apples of Sodom, Apples of Discord, Apples of the Eye. Nuts—Arom, Brazilian, Butternuts, Strych. Nux Vom.

ICES.

Cold Cream, Camphor Ice, Ice (Bags to the Spine).

CONFECTIONS.

Sugar Coated Pills, Tamarinds, Tropic Fruit, Chlor. Potass. Lozenges, Bronchial Troches.

DRINKS.

Sage-Tea, Bone-Set-Tea, Chamomile Tea, Whey, Coffee, with Glucose; Sugar of Milk, Sugar of Lead, Milk of Assafœtida, Condensed Milk, Goat's Milk, Cowmiss, Cream of Tartar.

Guests will be waited on by experienced nurses.—*The Cincinnati Lancet and Clinic.*

---

IS IT TRUE? HAS THE ILLINOIS STATE BOARD OF HEALTH UNCONDITIONALLY SURRENDERED TO COLUMBUS MEDICAL COLLEGE?—Such is the announcement made by *Gaillard's Medical*

*Weekly.* In October, Dr. Rauch, Secretary of the Illinois Board of Health, requested a formal communication from the West Virginia State Board of Health, giving its action respecting the Columbus Medical College. It was understood that it would sustain the West Virginia Board. But meantime a nephew of Dr. J. W. Hamilton, the owner of Columbus Medical College, had become governor of the State of Illinois. The Illinois State Board of Health has not sustained the West Virginia Board. By default, it recognizes the college which granted a diploma to a man after three weeks attendance upon a lecture course. Is this a stand that promises a higher medical education? Is this the Board which is to sweep Illinois free from uneducated doctors? Is three weeks lecture attendance sufficient to constitute a regular medical course? Alas, the times! the customs! It looks as if the owner of Columbus Medical College fixed his nephew, the governor of Illinois, so that the Illinois State Board dared not to peep against Columbus Medical College. Against little colleges which have little or no influence in the political circles regulating the appointing power of the Illinois State Board of Health, we may expect fulminations of excommunication, but the rest may do as they please. Such seems to be the lesson of the past, as regards this Board, and so it will be till the end of time. Political power regulating the practice of medicine is useless unless the people are themselves so educated that they can do their own regulating. Then State boards will not be needed. We are very sorry that the "back-bone" of the Illinois Board has proved to be "mush" against a really powerful and dangerour adversary. In fact, we hope that in some manner it may be shown that the whole affair is a myth and that the Board will sustain the action of the West Virginia Board. If not, the days of usefulness of the Board are ended.

---

THE PHILADELPHIA HOSPITAL FOR SKIN DISEASES, No. 923 Locust street, Philadelphia, Pa.

BOARD OF MANAGERS.—Robert E. Rogers, M. D.; Wm. H. Pancoast, M. D.; Rev. Geo. F. Wiswell, D. D.; C. W. McKeehan, Esq.; Geo. W. Fairman, Esq.; Hon. A. K. McClure;

Wm. S. Janney, M. D.; Wm. B. Atkinson, M. D.; Lawrence Wolff, M. D.; Geo. H. Brown, Esq. L. Wolff, M. D., Secretary.

ANNOUNCEMENT.

The Board of Managers of the Hospital for Skin Diseases, of Philadelphia, take pleasure in announcing that they have recently added to their facilities a complete system of baths for the general and specific treatment of this class of affections. Under this head are comprised the Turkish, Russian, vapor, medicated and electric baths, for the giving of which the latest and most approved apparatus has been obtained at considerable cost. The subject of balneology, as connected with skin diseases, is receiving much attention from specialists, and the physician in charge of the hospital is giving it the consideration that the importance of the subject demands. Peculiar advantages are thus afforded physicians and advanced students who wish to obtain a knowledge of this branch of therapeutics by visiting the hospital and consulting with the attending physician.

Free clinics are given at the hospital daily, at 11:30 a. m., by the physician in charge, Dr. John V. Shoemaker, where can be seen a great variety of diseases. The large variety of cases presented enable those who attend the clinics to gain in one or two courses a practical knowledge not obtainable in private practice. The course is made more comprehensive by didactic lectures which are delivered at the hospital on Mondays and Fridays, at 11 o'clock, A. M.

The dispensary is open for the reception of patients daily, Sundays excepted, at 11:30 A. M. Physicians and advanced medical students are always welcome, either as visitors or for the purpose of attending the free clinics. The superintendent, Mr. F. C. Waterman, will be in his office in the hospital every day from 11:30 A. M., to 2 A. M., to receive visitors or to transact business.

---

The following letter explains itself:

CLEVELAND, O., MAY 8, 1883.

MESSRS. EDITORS:—The next annual meeting of the American Medical Association will be held in this city, June 5th to

8th, inclusive. All railroads west of Pittsburg, Salamanca and Buffalo, east of Chicago and south of Cleveland, will carry delegates and members of their families to Cleveland at one full are, and return them on certificate signed by me as chairman of the committee (certifying that they have been in attendance at the meeting of the Association) at one cent per mile.

The trunk lines east of Buffalo, Salamanca and Pittsburg, have refused to make any reduction. The rates per diem at the hotels will be: Kennard House, $3; Weddell House, $3; Forest City House, $2.50 to $3; American House, $2.50; Hawley House, $2; Striebinger House, $2; Clarendon House, $2, and Prospect House, $2.

X. C. SCOTT,
*Chairman Committee of Arrangements.*

THE city Health Department reports for the month of April a mortality of 922. The highest numbers of causes reported as follows:

Phthisis pulmonalis, 93; pneumonia, 78; bronchitis, 53; diphtheria, 48; scarlet fever, 36; typhoid fever, 16; enteritis, 17; diseases of the heart, 31; infantile convulsions, 51; cerebral meningitis, 28; puerperal peritonitis, 4; peritonitis, 11; senile debility, 23; congenital debility, 20; still births reported, 64; 7 deaths from small-pox. Deaths under 5 years, 440; under 1 year, 209.

Comparative mortality. April, 1882, 1003; March, 1883, 968; April, 1883, 922.

Range of temperature, 28.3 to 78.3.

THAT quacks cannot be held in damages for malpractice is sustained by the following decision of an Oriental court. A fellow had a pain in his eyes, and went to a farrier, saying, "Give me medicine." The farrier applied to his eyes the remedies he was in the habit of using for his animals, and blinded him, on which he complained to the magistrate, who pronounced that he could not recover damages; "For," said he, "if this fellow had not been an ass, he would not have consulted a farrier."

FRIEDERICH thinks that in hysterical persons who practice masturbation, the clitoris is irritated by this manipulation and that the reflex symptoms, as convulsions, etc., are thereby produced. He has applied the nitrate of silver to the inflamed clitoris, and seen many cases where all severe symptoms were relieved after a few applications. He thinks further that the gynæcologist should not treat the uterine tract in hysterical persons, there being mostly no pathological phenomena.—*Memorabilien.*

PROF. JNO. E. OWENS has resigned the chair of Surgery in the Woman's Medical College.

AT a meeting of the Faculty of the Woman's Medical College, held at the Sherman House, May 17, Prof. D. W. Graham was elected to the chair of Surgery; Dr. Walter L. Dorland to the chair of Materia Medica and Therapeutics, and Dr. M. E. Bates to the lectureship of Anatomy.

PROF. T. DAVIS FITCH, of the Woman's Medical College, has been created Emeritus Professor of Gynæcology in that institution. His many friends will be pleased to hear of his satisfactory progress toward recovery, and his ability to again resume his practice.

Prof. M. J. Mergler has been elected associate Professor of Gynæcology.

BEGINNING with the first number of the tenth volume, issued May 3, the *Maryland Medical Journal* was changed to a weekly publication, and will appear every Thursday. Each number will contain sixteen pages, double column, of solid reading matter. The size and appearance of the *Journal* will be slightly altered to conform to the requirements of a weekly.

TREATMENT OF GRANULAR CONJUNCTIVITIS BY SEQUERITI.

Sequeriti is universally applied in diseases of the eye in Brazil. The remedy has been tried in Paris with apparently good results. The seeds of the plants are boiled in water (1:100), and the inflamed lids washed with the solution three times a day.—*Le Praticien.*

